# Large language models in physical therapy: time to adapt and adept

**DOI:** 10.3389/fpubh.2024.1364660

**Published:** 2024-05-24

**Authors:** Waqar M. Naqvi, Summaiya Zareen Shaikh, Gaurav V. Mishra

**Affiliations:** ^1^Department of Interdisciplinary Sciences, Datta Meghe Institute of Higher Education and Research, Wardha, India; ^2^Department of Physiotherapy, College of Health Sciences, Gulf Medical University, Ajman, United Arab Emirates; ^3^NKP Salve Institute of Medical Sciences and Research Center, Nagpur, India; ^4^Department of Neuro-Physiotherapy, The SIA College of Health Sciences, College of Physiotherapy, Thane, India; ^5^Department of Radiodiagnosis, Datta Meghe Institute of Higher Education and Research, Wardha, India

**Keywords:** artificial intelligence, BioMedLM, evidence-based practice, large language models, physical therapy, physical therapy education, rehabilitation

## Abstract

Healthcare is experiencing a transformative phase, with artificial intelligence (AI) and machine learning (ML). Physical therapists (PTs) stand on the brink of a paradigm shift in education, practice, and research. Rather than visualizing AI as a threat, it presents an opportunity to revolutionize. This paper examines how large language models (LLMs), such as ChatGPT and BioMedLM, driven by deep ML can offer human-like performance but face challenges in accuracy due to vast data in PT and rehabilitation practice. PTs can benefit by developing and training an LLM specifically for streamlining administrative tasks, connecting globally, and customizing treatments using LLMs. However, human touch and creativity remain invaluable. This paper urges PTs to engage in learning and shaping AI models by highlighting the need for ethical use and human supervision to address potential biases. Embracing AI as a contributor, and not just a user, is crucial by integrating AI, fostering collaboration for a future in which AI enriches the PT field provided data accuracy, and the challenges associated with feeding the AI model are sensitively addressed.

## Introduction

**Figure 1:**
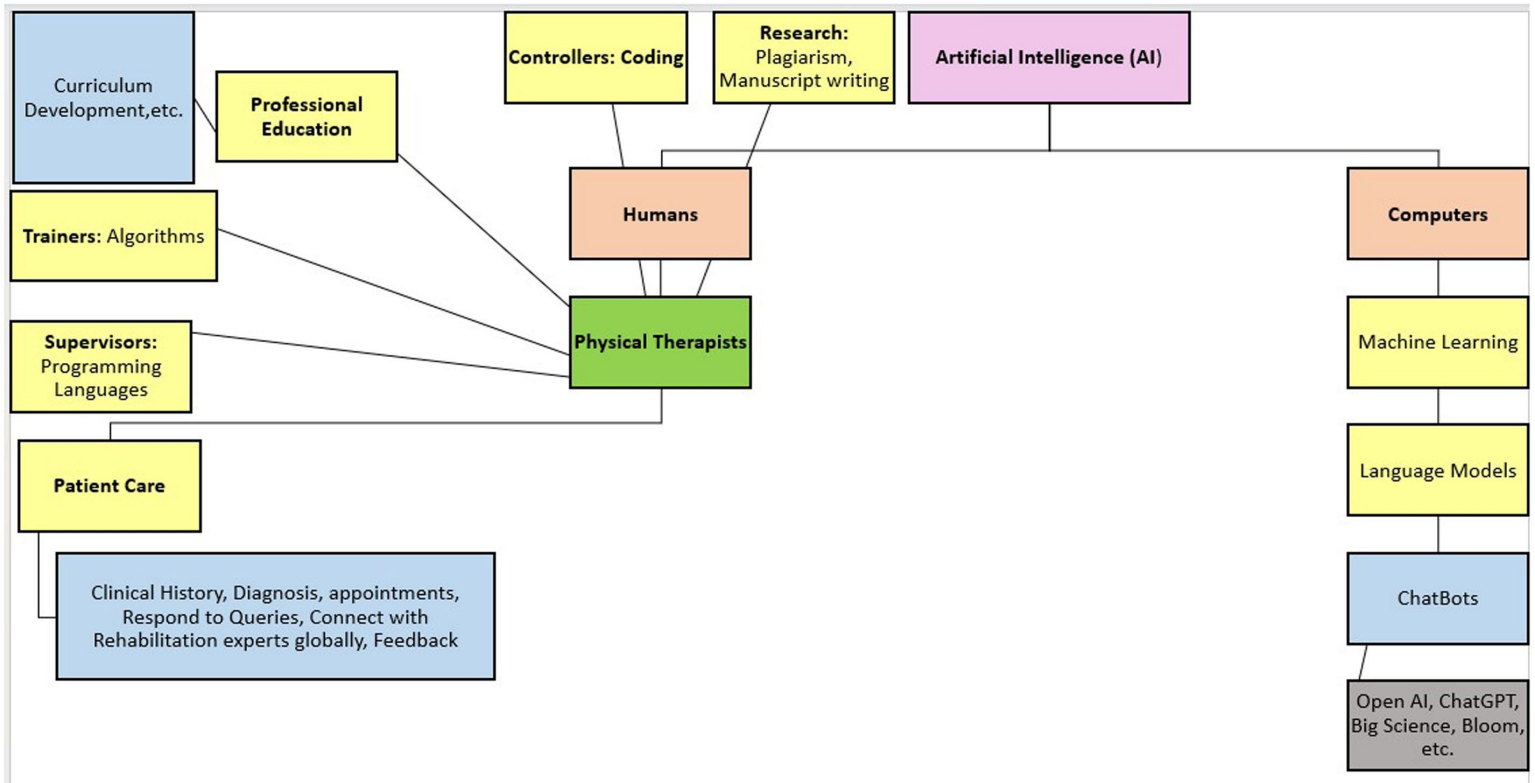
Overview of utility of artificial intelligence in physical therapy.

The healthcare landscape is undergoing a seismic shift driven by the relentless advancement of artificial intelligence (AI) and machine learning (ML) (1). Physiotherapists, like all healthcare professionals, stand at the precipice of this transformative era in professional education, clinical practice, and research. While some may view AI as a threat, it presents a unique opportunity for physiotherapists to elevate our practice and revolutionize patient care (2). Computer programs that indirectly enable humans to seem intelligent by doing tasks, providing options, and executing decisions on behalf of humans are called artificial intelligent systems (3) (Figure 1). Chat Generative Pre-trained Transformer (Chat-GPT) has managed to achieve human-like performance using deep ML and deep neural networks (4). A large amount of complex data is collected in the medicinal field, which often requires analysis to break down complex information into simple interpretations. Although OpenAI’s ChatGPT is one of the biggest LLM marching ahead in AI dataset development and accuracy, due to the presence of vast data, it gets difficult to have accurate results on ChatGPT (5). Stanford University is among the pioneers in developing BioMed-LLM focused on datasets created from the PubMed database. There are also other LLMs that are more focused on medical databases and datasets, such as Microsoft’s Bio-GPT and Google’s Med-PaLM (6). These datasets are in the stage of fine-tuning to provide more accurate and proficient results. The LLM programs enable the clinician, medical researcher, and educator to make scientifically informed decisions with respect to symptoms, assessment, diagnosis, and further plans of action (7).

*Generative AI* is an umbrella term used when AI is used to generate ideas, content, images, etc. It is defined as a branch of ML that translates innovative content in the form of text, images, and audio-visual into computer codes (8). Generative AI ranges of standardized assessments in medical education not only underscore their capabilities but also prompt a reconsideration of our existing evaluation methods (9). Generative AI delves into the burgeoning field of generative AI and its applications within the medical domain. Mohammed H. M. Amin et al. in their study, explore the utility of generative adversarial networks (GANs) for brain tumor segmentation, demonstrating its potential in enhancing medical image analysis tasks. Kumar, Sharma, and Tyagi provide an extensive overview of the state-of-the-art and recent developments in GANs, shedding light on their diverse applications and promising advancements. Thakkar, Anand, and Jaiswal discuss the burgeoning applications of GANs in healthcare, highlighting their significance in various medical contexts. Fasakin et al. conducted a comprehensive survey on the utilization of GANs in healthcare, presenting an analysis of trends, techniques, and applications, offering valuable insights into the field’s current landscape. Finally, Paul, et al. focus on medical image synthesis using GANs, showcasing their efficacy in generating synthetic medical images, thereby facilitating research, training, and diagnostic tasks in the medical domain. These articles collectively underscore the growing significance of generative AI in revolutionizing medical imaging, diagnostics, and healthcare delivery.

There are other types of AI that group, assign, choose, and decide, and can be semi to fully automated as well. Generative AI systems have been developed for content creation, idea generation, image creation, and audio creation. Overall, generative AI terminology has been utilized depending on the type of usage by consumers. There are, however, certain support tools or systems developed which are related to *language*. The way coding programs are used for the development of application-based technology, similarly, language coding is used as a support tool by researchers and educators for creating academic content for AI chatbot systems (10). The LLM would serve as a tool to assist physical therapists (PTs) in effectively using the capabilities of generative AI. Our paper contributes to a general discussion among research groups worldwide, necessitating the use of Generative AI and LLMs in physical therapy and rehabilitation. To conclude, large language models (LLMs) are programs developed in order to simplify complex content by providing a comprehensive summary (11, 12). However, the question arises: Is the content useful, valid, reliable, and scientific? To answer this question: yes, it can be all of the above, provided humans feed into this system of AI, although it is full of challenges and limitations, and ensuring strict protocols to train the LLM of relevant literature databases for training and utilization. It is also essential that the LLMs be fed with unbiased, factually accurate data. Literature in the past 5 years states that the application of LLMs in the realm of healthcare, including areas such as neurology, oncology, psychiatry, and more, has shown promising results, except for studies directly pertaining to physical therapy, which are limited in the current research data (Table 1).

**Table 1:** Background review of literature on the current utility of AI in healthcare.

Title	Objective	Methodology	Key findings
Recognizing physiotherapy exercises using machine learning (13)	Developed a system for Physiotherapy exercise assessment using pose detection.	Uses pose detection for exercise assessment with a camera and provides feedback on posture and body angles.	96% accurate for pose detection.
Artificial intelligence and machine learning applications in musculoskeletal physiotherapy (14)	Utility of machine learning (ML) in musculoskeletal medicine.	Review on supervised and unsupervised ML with diagnostic imaging and clinical decision-making assistance.	ML can enhance physiotherapy practice through the automation of tasks involving data analysis, classification, and prediction.
Artificially Intelligent Physiotherapy (15)	Framework using AI and ML for digitalized physiotherapy practice.	Uses OpenPose Library for detecting joint angles and providing exercise suggestions based on performance.	Enables remote physiotherapy sessions with personal feedback, aiming for cost reduction.
Machine Learning Approach for Physiotherapy Assessment (16)	Enhance the accessibility of physiotherapy treatment using a Kinect sensor and inertial measurement unit (IMU) for motion tracking.	Tracks human motion using a Kinect sensor and IMU chip, with a feedback band for movement correctness.	Framework supports remote physiotherapy sessions, reducing treatment costs.
Automated Error Detection in Physiotherapy Training (17)	Develop a system for scoring and feedback in manual skills training using sensor data.	Utilizes IMU and sEMG sensor data for gesture classification, scoring, and feedback	90% accurate in movement pattern execution for manual skills training.

### Ensuring data accuracy and challenges

Data accuracy stands as the cornerstone of reliable and impactful decision-making. However, as AI systems become more sophisticated, several challenges emerge in ensuring the accuracy of the data they process based on four major challenges (18, 19):

- Model Forgetfulness: It is a tendency of AI models to gradually lose previously acquired knowledge over time, particularly when exposed to evolving datasets, especially when it is supposed to predict or forecast something.
- User Acceptability: It is the willingness of end-users to trust and use AI-driven solutions in their decision-making, depending on the transparency of the LLMs and its features.
- Explainability: The ability of AI systems to provide interpretable explanations for their decisions and predictions, often referred to as “black boxes.”
- Hallucination effect: It occurs in generative AI models where the system generates false or unrealistic outputs that resemble real data, which poses risks in applications such as image generation, text synthesis, and data augmentation.

**Figure 2:**
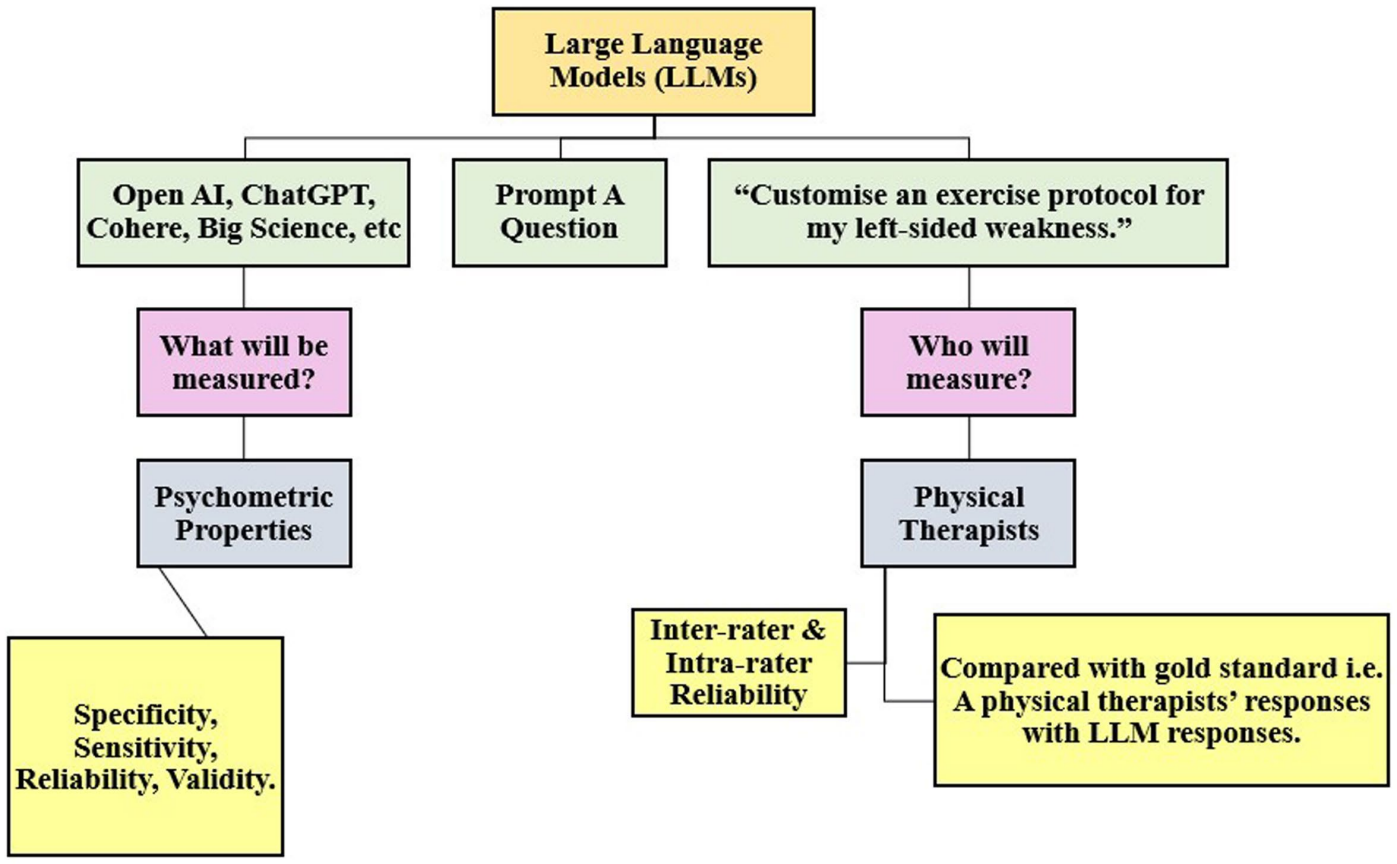
Processing steps of a large language model (LLM).

**Figure 3:**
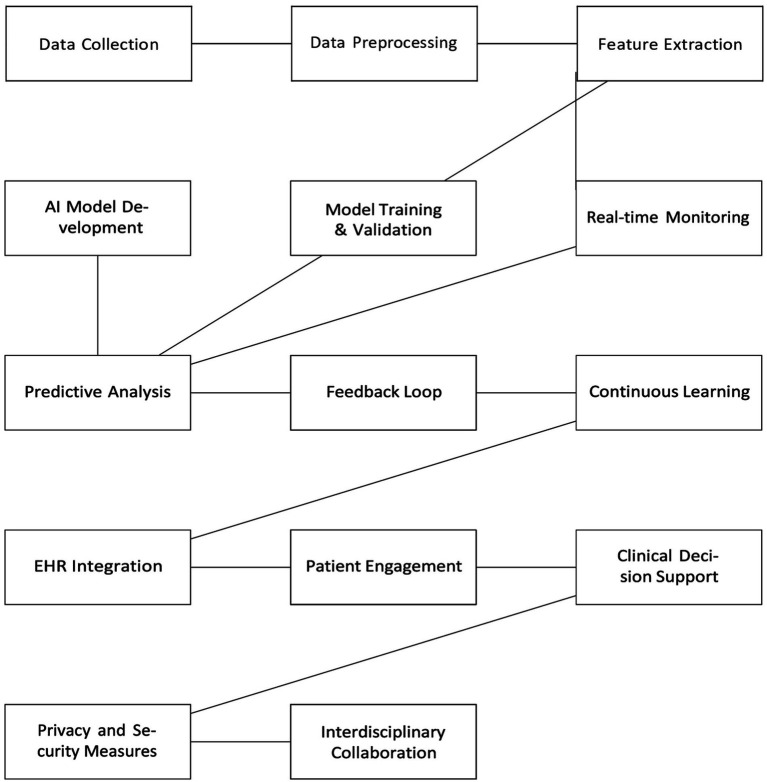
Schematic diagram of embracing al in physiotherapy applications. *EHRs: Electronic health records.

Humans will play the role of creators, and supervisors and their responsibility would be to ensure setting up boundaries for judicious use of these models and programs. The other factor deciding the reliability of content would be the process involving data management, data filtering, and the level of training provided to these programs and users for ethical use of the system. The pros definitely outweigh cons of AI, although the cons can pose a serious risk if not taken into account. The potential benefits of AI and ML in physical therapy are vast and encompass several crucial areas (Table 2). Medical and health sciences practitioners and educators have delved into the use of language models in ophthalmology, radiology, orthopedics, dermatology, surgery, obstetrics, and gynecology for educational and clinical practice development (20–24). Wilhem et al. evaluated the performance of four language models, i.e., GPT-3.5. Turbo, Bloomz, BigScience, among others, created medical content across the above specialties and concluded that LLMs are capable of generating consistent and trustworthy, clinically safe medical advice to specific prompts (25). Studies by Mesko et al. conclude that content creation is consistent and trustworthy, and LLM-generated medical advice can be tailored to their cases based on clinical guidelines and expert opinions. However, limited studies have validated the consistency of responses to similar prompts or case scenarios to assess the robustness of LLMs and their efficacy. The steps ideally include prompting, screening by an expert, review of responses by another set of experts, fine-tuning of errors, and benchmarking them against gold standard datasets to assess user experience and feedback. In this way, LLM responses can be modulated and compared after being fed with accurate data and evidence-based clinical practice guidelines to meet patient needs (26). A recent scoping review by Ullah et al. assessed the challenges and barriers to using LLMs in diagnostic medicine in the field of pathology (27). Many language models, such as Claude, Command, and Bloomz, have been programmed for creating accurate medical advice (28) (Figure 2). Hence, recognizing AI as more than just a tool but as a genuine contributor is essential, which entails integrating AI into physical therapy to improve data accuracy and encourage collaborative efforts. It is crucial to address the challenges associated with training AI models sensitively, particularly handling patient data and upholding ethical standards. Therefore, navigating these obstacles thoughtfully, we can completely leverage AI’s potential to revolutionize the practice of physical therapy (Figure 3).

**Table 2:** Advantages of large language models in clinical practice, professional education, and research.

A. Clinical practice: Patient assessment and screening. Customization of tailor-made rehabilitation protocols. Patient counseling and education. Accurate diagnosis. B. Professional education: Development of learning resources for students. Curriculum development and enhancement. Quality assurance. Creating examination formats (e.g., short answers, quiz, and multiple choice questions) C. Research and evidence-based practice: Literature review Research synthesis Plagiarism detection Referencing Integration of data into manuscript publication.

The perspective discussed in this paper is how and why language learning models (LLMs) of the AI universe will be a sweeping game-changer in physical therapy in terms of professional education, clinical practice, and evidence-based practice.

### Training of models by physical therapists

- *Why to train:* LLMs are large-sized programs of the AI system that have been fed into datasets and trained by infinite words, bibliography which have been imported from scientific articles, books, and other content which is available on the internet search engines (29). For example, if the LLM system is developed in such a way that only PubMed database articles, books, and other literature is needed from a particular database. This was established by Stanford University in which they developed a chatbot LLM of 2.7B in size extensively trained and imported from the PubMed database for answering model questions and answers, which was previously known as PubMed GPT in the field of medicine website link (30). On similar lines, in nursing education, Glauberman et al. have concluded that algorithm biases must be addressed at each step of training the LLM with a continuous evaluative process, and data of many different races, conditions, and severity must be incorporated and categorized (31). AI diminishes humans’ capacity to learn from our experiences; hence, these experiences must be coded, reviewed continuously, and fed, and re-fed with ever-changing factual data by human experts at multiple levels (32) *How to train:* Deep ML is a method of training the machine or programs with many layers. For instance, processing large amounts of images, videos, and written content across a particular field such as physical therapy practice, education, and research. Data management must be trained by data managers via standard and enriching meta-data according to FAIR guidelines (Findable, Accessible, Interoperable, Reusable) (33). Human interruption or supervision, is essential at every step. It has to be trained by humans efficiently in order to be used by humans, as it augments our work more efficiently. Humans will not be replaced or else the risk of automation is huge (34).
- *What to train:* Data feeding into these systems can be in several formats and types. Most commonly evaluated cases in musculoskeletal rehabilitation, such as degenerative, inflammatory, and so on, anatomy of the involved joints, specific pointers in history taking, differential diagnosis, specific investigations required, patient education, the acute and chronic line of treatment, time required to assess, diagnose, and complete the treatment and much more, can be entered into the LLM with strong, scientific evidence (35). On the other hand, the system can be linked with high-quality databases and journals, which will be qualified based on their journal metrics, type of peer review, and other such factors to enable the facilitation of high-quality content and can be proven as a verified source of information (36). Similarly, this format can be applied to various branches of physical therapy rehabilitation such as neurological, pediatrics, geriatrics, cardiovascular, pulmonary, community-based, hand, and sports (37). All of these can be categorized and classified according to the International Classification of Function (ICF) domains and processed in a uniform system by the ICF codes (38, 39).
- *Whom to train:* Not only the program developers and bio-engineers but also the PTs should start learning coding, algorithms, and types of AI, its chatbots, language models, and its basic functioning. There are also no coding applications, such as visual studio code (VSC), a free support tool application that can be of great help in debugging, refining, and editing web applications. It is the above stakeholders who, in turn, can train the language model systems for developing reliable, valid, sensitive, and specific responses to the prompt or question asked by the client (40).
- *Levels of training:* The training has to be extensive, meaning it should be part of the curriculum where the stakeholders can receive formal training hours under expert guidance in the domains of education, examination, administrative, clinical, and research. Inter-rater and intra-rater reliability will be bi-directional and calculated in two ways. First, there will be human prompts, and second, there will be machine prompts. Similar methods would be applied for machine and human responses. The process of establishing the validity of AI will be similar to validating a new instrument, technique, or method. The gold standard validator will be humans. The responses of the machine must be validated according to face, content, and design with the responses of humans, especially physical therapy experts (41).
- *Will the process be smooth or not?* The smoothness of the process will depend on the type of language models, user interface, user experience, recall value, and most specifically, the feedback of the patient or client. The entire process should be competent enough in terms of patient engagement, patient recall, patient’s user experience, satisfaction, and how truthful, harmless, and hassle-free the processes were. However, feedback from the client at every step becomes imperative (42).
- *Regulation and Recognition by the Physiotherapy organizations globally:* In the meantime, we can predict that AI, its LLMs, its types, and variants will be part of the curriculum recognized by universities, organizations, research institutions as well as physical therapy licensing authorities, associations, and societies for uniform ethical and considerate use worldwide (43, 44).
- *How will it be regulated?* Global accreditation of these LLMs must be regulated by a stringent screening system to ensure malpractices, unethical usage, and plagiarism are avoided at the grassroot level by the above regulating authorities. This will also aid in minimizing plagiarism and unethical misconduct and make stakeholders wary of falling into the trap of predatory influences (45).

### A call for the physical therapists

The burning question is: will LLMs achieve the desired quality in comparison to human touch? The answer to it is no, as human touch can never be replaced, mimicked, or be imitated. However, it can help a PT significantly by mechanizing other jobs and do it in a much better way than humans (46). Additionally, AI appears to be a current deficit in the medical curriculum, and most students surveyed were supportive of its introduction. These results are consistent with previous surveys conducted internationally (47). Rowe et al. have summarized on how AI can replace PTs by minimizing the extra effort the therapists put and maximizing their output with more objectivity. They also conclude that automation of tasks can be a matter of concern if not guided by ethical principles (48). Supervision and review of PTs become imperative, for example in diagnostic imaging, patient measurement data, and clinical decision support. Tack concludes that the existing literature base can identify cases as a preliminary step where ML is capable of performing equal to or more accurately than human levels in the field of physical therapy (14). Interacting with a patient at first go, engaging the client, taking notes, documenting, taking history, differentially diagnosing the patient’s symptoms, patient education and counseling, connecting with the best experts globally without leaving the comfort of your home, scheduling the appointments, analyzing movement, function, restrictions, and customizing a tailor-made treatment protocol in accordance with smart goals can prove to be a boon to PTs saving them time, effort, and money and providing a chance to treat a larger number of patients in a particular time frame (49). Overall, this will engage the client in a much more effective way, maintain communication standards, prevent cumbersome visits to treatment centers, and maximize patient follow-up. Valuable time, money, and energy will be saved and the client can be given the option to make scientifically informed decisions with active participation in the treatment (50) (Table 3).

**Table 3:** Ways how LLMs can reduce a physical therapist’s workload.

Physical therapist-related	Client-related
1. Less time consuming	1. Client engagement and satisfaction
2. Differential diagnosis based on symptoms	2. History taking
3. More clients can be assessed in a given time period.	3. Making notes
4. Workload reduction in documentation	4. Documentation
5. Comprehensive and evidence-based review of literature.	5. Scheduling appointments
6. Uniform system of curricular assessment and evaluation.	6. Suggest experts globally across specialties
	7. Protocol designing
	8. Client follow-up with high recall
	9. Saves effort, money

The physical therapy curriculum differs worldwide in terms of content, subjects, credit hours, nomenclature of the degree, course duration, course outcomes, evaluation methods, basis of evaluation, and types of projects to be completed, even though all PTs treat and assess same conditions and diseases in any corner of the world. Culminating these variations and discrepancies into one is only possible from developing and using a single, concrete type of unified system accessible to all and trained uniformly. The medical education field can hugely benefit from creating common examination patterns globally. The recent success of LLMs, such as BioMedLM, scoring exceptionally high on mock United States Medical Licensing Exams (USMLEs), highlights the immense potential of AI in healthcare (51). When a formally trained licensed PT can treat any client in the world, these variations in training, licensing, and practice must be bonded with a common thread or a language. Therefore, it necessitates the need to develop a common scientific curriculum and assessment protocol for the benefit of the PT’s community. These protocols must be error-free, continuously updated, and flexible and competent enough to adapt with continuous feedback assessment (52). Current challenges are listed in Table 4, along with strategies to address challenges associated with implementing generative AI in physical therapy while fostering acceptance and collaboration within the medical community and among patients and users.

**Table 4:** Current challenges and strategies for future directions in physiotherapy.

Challenges	Strategies
Data availability and quality	1. Collaborate with healthcare institutions for access to diverse and high-quality datasets.
	2. Implement data augmentation techniques to expand dataset diversity.
	3. Ensure data privacy and security measures are robust.
Implementation frameworks	1. Develop standardized protocols for integrating generative AI into physical therapy practices.
	2. Provide training and resources to PT professionals for seamless integration.
	3. Foster interdisciplinary collaborations with AI experts.
Model evaluation	1. Establish comprehensive evaluation criteria, including clinical efficacy and safety.
	2. Utilize cross-validation techniques to assess model performance.
	3. Continuously monitor and update models based on real-world feedback.
Policy and regulatory challenges	1. Advocate for clear guidelines and regulations tailored to AI applications in healthcare.
	2. Collaborate with policymakers and regulatory bodies to ensure compliance.
	3. Stay informed about evolving legal frameworks and adapt accordingly.
Biases	1. Conduct thorough bias assessments during model development.
	2. Implement techniques such as fairness-aware learning to mitigate biases.
	3. Regularly audit AI systems for bias detection and correction.
Patient and community acceptance	1. Educate patients and communities about the benefits and risks of AI in physical therapy.
	2. Solicit feedback and involve stakeholders in decision-making processes.
	3. Maintain transparency regarding AI usage and outcomes.

Therefore, it becomes necessary for PTs to learn language models that will enable the amalgamation of technology, ML, and the rehabilitation field. Coding must be a part of the curriculum to build and design efficient physical therapy LLMs in clinical, educational, and research settings among health professions education to harness the complete potential of AI and ML requires proactive engagement from physiotherapists (53). We must embrace AI and ML, explore the capabilities of these technologies, and identify opportunities for integrating them into our practice. Start small, experiment, and learn from experience to become familiar with BioMed-LLMs and Biomed Natural Language Processing (NLP) (54). These support tools are crucial for navigating the vast medical literature, extracting valuable insights and mastering Biomed-NLP allows one to process and interpret complex medical information, enhancing one’s understanding and decision-making by contributing to dataset *fine-tuning*, our expertise will become invaluable in ensuring that AI and ML models are trained on accurate and scientifically transparent data (55, 56). We, as a community, can also ensure that these language models are optimized for the specific needs of physiotherapy practice in rehabilitation. Collaborating with researchers and developers to create AI and ML tools specifically designed for physiotherapy will directly influence the development of applications and support tools to address unique challenges faced by physiotherapists (57). A collaborative environment can be promoted that fosters innovation and the adoption of AI and ML technologies by sharing our experiences, knowledge, and insights, thus supporting our colleagues in their learning journey. Although these models pose challenges such as bias and explainability, they offer a glimpse into the future of medical education and evaluation, where AI will act as a powerful tool to enrich learning and assessment for medical and allied health sciences curriculum and practice (58).

## Limitations

Majovsky et al. conducted a study that evaluated the generation of an authentic-looking fabricated scientific paper using ChatGPT and that is possible but human supervision is a mandate (59). Plagiarism detection software and ethical considerations must align with the updated ICMJE recommendations for using AI technology. AI detection tools should be employed to screen and negotiate content, and stringent boundaries must be established. The future trajectory of AI will heavily depend on how these ethical challenges are addressed (60).

This paper is a call for action by physiotherapists to embrace AI and ML by acquiring necessary knowledge and actively shaping their development, we can ensure that our profession remains at the forefront of healthcare in this evolving landscape. We have the opportunity to revolutionize physical therapy and improve patient care. We authors feel this is an apt time and opportunity for PTs to delve into LLMs, not only to utilize it but also to learn to develop and fine-tune the model for a better future. Etymologically speaking, PTs must take charge as experts, i.e., *adept* by being proficient rather than adapting to changing times in the coming decade. Let us consider this opportunity and lead the way to the future of AI-driven healthcare by doing more collaborative studies, creating new beginnings with an admixture of various disciplines. It is time to delve into LLM training, fine-tune the datasets, and provide solutions at par with those of other medical and health sciences professionals for the future. *Language Model* programming in AI is the future of medical and allied health professions, especially physical therapy.

## Data availability statement

The raw data supporting the conclusions of this article will be made available by the authors, without undue reservation.

## Author contributions

WN: Conceptualization, Writing – original draft, Writing – review & editing. SS: Writing – original draft, Writing – review & editing. GM: Writing – review & editing.
